# Cutaneous wound healing promoted by topical administration of heat-killed *Lactobacillus plantarum* KB131 and possible contribution of CARD9-mediated signaling

**DOI:** 10.1038/s41598-023-42919-z

**Published:** 2023-09-23

**Authors:** Shinyo Ishi, Emi Kanno, Hiromasa Tanno, Shiho Kurosaka, Miki Shoji, Toshiro Imai, Kenji Yamaguchi, Kanna Kotsugai, Momoko Niiyama, Haruko Kurachi, Fuko Makabe, Takumi Watanabe, Ko Sato, Keiko Ishii, Hiromitsu Hara, Yoshimichi Imai, Kazuyoshi Kawakami

**Affiliations:** 1 Department of Plastic and Reconstructive Surgery, Tohoku University Graduate School of Medicine, 2-1 Seiryo-cho, Aoba-ku, Sendai, Japan; 2 Department of Translational Science for Nursing, Tohoku University Graduate School of Medicine, 2-1 Seiryo-cho, Aoba-ku, Sendai, Miyagi 980-8575 Japan; 3 Present Address: Bio-Lab Co., Ltd, 2-1-3 Komagawa, Hidaka-shi, Japan; 4 Department of Medical Microbiology, Mycology and Immunology, Tohoku University Graduate School of Medicine, 2-1 Seiryo-cho, Aoba-ku, Sendai, Japan; 5 Department of Intelligent Network for Infection Control, Tohoku University Graduate School of Medicine, 2-1 Seiryo-cho, Aoba-ku, Sendai, Japan; 6 Present Address: Department of Clinical Microbiology and Infection, Tohoku University Graduate School of Medicine, 2-1 Seiryo-cho, Aoba-ku, Sendai, Japan; 7 Department of Immunology, Graduate School of Medical and Dental Sciences, Kagoshima University, Kagoshima, Japan

**Keywords:** Acute inflammation, Biological therapy

## Abstract

Optimal conditions for wound healing require a smooth transition from the early stage of inflammation to proliferation, and during this time alternatively activated (M2) macrophages play a central role. Recently, heat-killed lactic acid bacteria (LAB), such as *Lactobacillus plantarum* (*L. plantarum*) have been reported as possible modulators affecting the immune responses in wound healing. However, how signaling molecules regulate this process after the administration of heat-killed LAB remains unclear. In this study, we examined the effect of heat-killed *L. plantarum* KB131 (KB131) administration on wound healing and the contribution of CARD9, which is an essential signaling adaptor molecule for NF-kB activation upon triggering through C-type lectin receptors, in the effects of this bacterium. We analyzed wound closure, histological findings, and inflammatory responses. We found that administration of KB131 accelerated wound closure, re-epithelialization, granulation area, CD31-positive vessels, and α-SMA-positive myofibroblast accumulated area, as well as the local infiltration of leukocytes. In particular, M2 macrophages were increased, in parallel with CCL5 synthesis. The acceleration of wound healing responses by KB131 was canceled in CARD9-knockout mice. These results indicate that the topical administration of KB131 accelerates wound healing, accompanying increased M2 macrophages, which suggests that CARD9 may be involved in these responses.

## Introduction

Skin wound healing is a process involving complex interactions among various types of cells and progresses through stages characterized by inflammation, proliferation, and remodeling. Among these, a smooth transition from the early stage of inflammation to proliferation is a critical step in wound healing^1^. The inflammatory response leads to the secretion of several cytokines and chemokines^2^ that are involved in the cellular migration necessary for the transition to the next remodeling phase.

Many types of cells are involved in the healing process, and in particular, macrophages are well known as pivotal players that enhance the transition of proliferative reactions in wound sites. Macrophages are commonly grouped into two distinct subsets affected by environmental factors, namely, classically activated (M1) macrophages and alternatively activated (M2) macrophages. The former is involved in proinflammatory responses and the latter contributes to anti-inflammatory and proliferating responses by producing anti-inflammatory cytokines and growth factors such as TGF-β^3–5^. Thus, it was suggested that M2 macrophages are the central regulators of proliferating responses in the wound healing process^6^.

Several species of viable lactic acid bacteria (LAB), including *Lactobacillus plantarum* (*L. plantarum*), are well known to have beneficial effects on tissue remodeling, such as keratinocyte migration^7^ and regulating immune responses at the wound sites^8,9^. It was reported that *L. plantarum* whole cultures promoted tissue repair^9^, and this bacterium may also improve the healing of diabetic wounds in rats through the regulation of inflammatory cytokines^10^. There are safety concerns related to the risks of using live microorganisms, however, such as systemic infections and excessive inflammatory responses^11,12^. Against this background, recent research has focused on the contribution of heat-killed LAB to wound healing^13–15^. It has been reported that heat-killed *Enterococcus faecalis*^13^, *L. plantarum* GMNL 6 and *L. paracasei* GMNL 653^14^, or *Lactococcus chungangensis* CAU 1447^15^ leads to improved wound healing in skin. Tsai et al. reported that heat-killed *L. plantarum* GMNL 6 promotes collagen synthesis in Hs68 human fibroblast cells (in vitro), and accelerates the wound healing of mice tail wounds^14^. However, it remains to be elucidated how heat-killed LAB affects the kinetics of M1 and M2 macrophage accumulation at wound sites and how signaling molecules modulate the healing process promoted by the administration of heat-killed LAB.

Heat-killed LAB contains several pathogen-associated molecular patterns (PAMPs), such as components of the cell wall^16^ and extracellular polysaccharides^17^, which were reported to induce immune responses. These molecules contribute to the upregulation of inflammatory responses through NF-kB activation by binding to pattern recognition receptors (PRRs), such as Toll-like receptors (TLRs)^16^ and C-type lectin receptors (CLRs)^18^, which are mainly expressed on macrophages. As for the role of CLRs, we previously showed that CARD9^19^, an essential signaling adaptor molecule through CLRs, including Dectin-1 and Dectin-2^20,21^, plays a key role in wound healing.

Against this background, in the current study, we conducted analyses to define the effects of topical administration of heat-killed *L. plantarum* KB131 (KB131) on wound healing, with a particular emphasis on the kinetics of the accumulation of macrophage subtypes and the involvement of CARD9 in these responses. *L. plantarum* is well documented for its safety, offering diverse applications. This bacterium is found in various environments such as in dairy products, pickled vegetables, fish products, and mammal intestinal tracts^22^. Here, we demonstrated that the topical administration of KB131 led to the acceleration of wound healing and the accumulation of M2 macrophages from the early phase to the proliferative phase and identified the possible contribution of CARD9 to the regulation of this healing process.

## Results

### Accelerated skin wound healing by topical administration of heat-killed *L*. *plantarum* KB131

**Figure 1:**
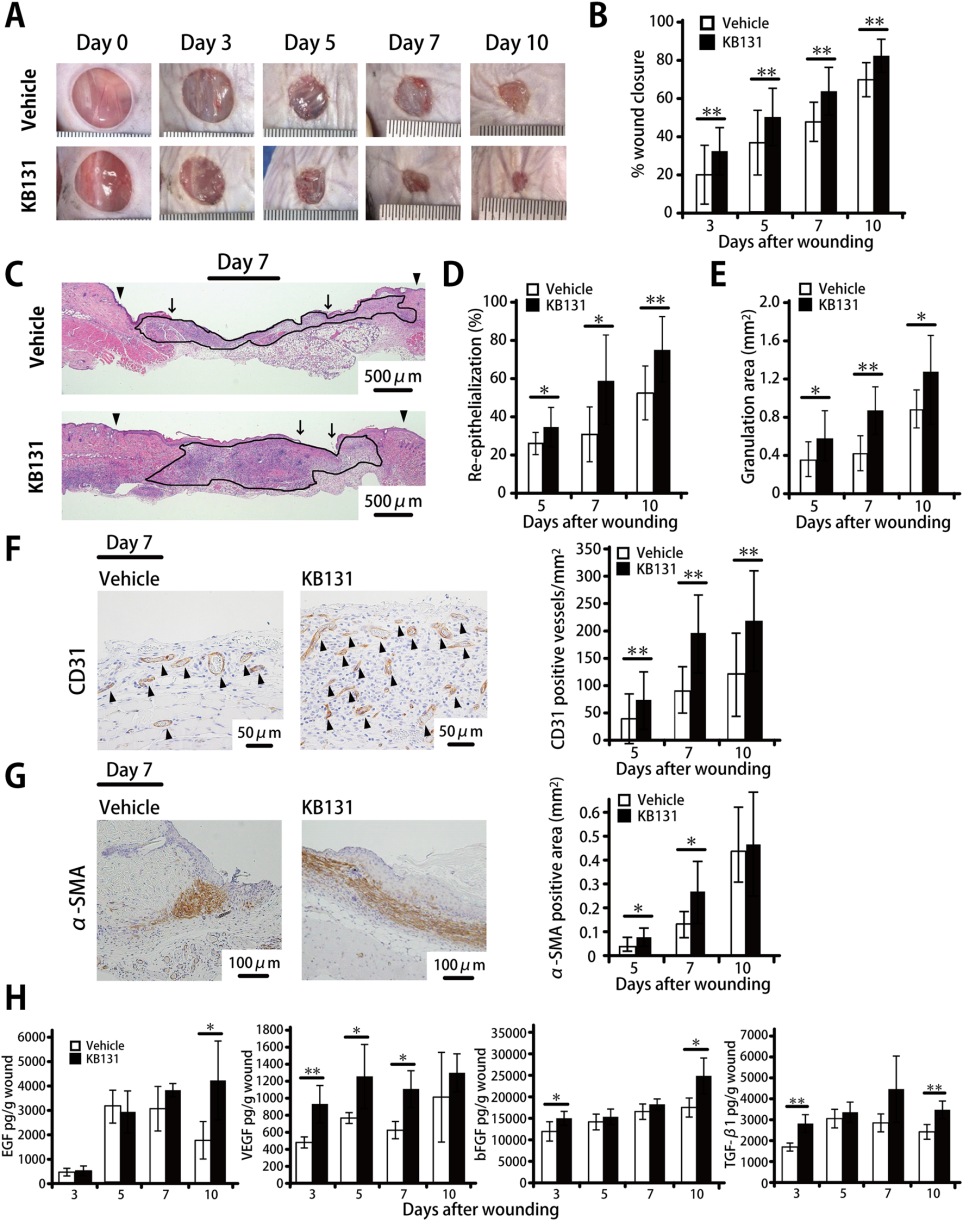
Effects of heat-killed KB131 treatment on wound healing. Four full-thickness wounds were created on the backs of WT mice. Immediately after wounding, a 5-μL suspension of heat-killed *L. plantarum* KB131 or vehicle control (distilled water) was applied to the base of the wounds. (**A**) Representative photographs of wounds on days 0, 3, 5, 7, and 10. (**B**) Percentage of wound closure was evaluated on days 3, 5, 7, and 10 (n = 20 wounds of five mice per group). (**C**) Representative histological views of the skin wounds on day 7 are shown. Arrowheads and arrows indicate the re-epithelialized leading edges and the original wound edges, respectively. (**D**) The re-epithelialization ratio on days 5, 7, and 10 (n = 10 wounds of five mice per group). (**E**) Time-course changes in the granulation tissue area per mm^2^ on days 5, 7, and 10 after wounding (n = 10 wounds of five mice per group). (**F**) Representative histological views of the skin wounds on day 7 are shown. The number of vessels stained with anti-CD31 antibody on days 5, 7, and 10. Arrowheads indicate CD31-positive vessels. The vascular density per mm^2^ was determined by counting the positive vessels (n = 10 wounds of five mice per group). (**G**) Representative histological views of the skin wounds on day 7 are shown. Time-course changes in the myofibroblast accumulated area per mm^2^ stained with α-SMA antibody on days 5, 7, and 10 (n = 10 wounds of five mice per group). (**H**) EGF, VEGF, bFGF, and TGF-β1 levels in the wounded tissue homogenates were measured at days 3, 5, 7, and 10. Four wounds were created in one mouse, which were combined into one sample, and five mice were analyzed in each group (n = 5 mice per group). Each column represents the mean ± SD. *p < 0.05, **p < 0.01. *α-SMA* α-smooth muscle actin, *h* hour, *KO* knockout, *mm* millimeter, *WT* wild type.

To elucidate the influence of KB131 on wound healing, we analyzed the effects of the topical administration of this bacterium at the wound sites. In our pilot experiments (data not shown), KB131 showed the highest acceleration of wound healing at a dose of 125 μg/wound compared with the other doses (1.25 or 12.5 μg/wound), and we confirmed that similar levels of cytokines were produced by spleen cells upon stimulation with viable or heat-killed KB131. Therefore, in this study, we analyzed the effects of 125 μg KB131/wound. In mice treated with KB131, wound closure was significantly accelerated at days 3, 5, 7, and 10 compared to mice treated with vehicle control (Fig. 1A,B). Moreover, the re-epithelialization rate and granulation area were found to be significantly increased in the KB131-treated group compared with mice treated with vehicle control (Fig. 1C–E). As alternate indicators of skin wound healing, we evaluated CD31 and α-SMA, which indicate neovascularization and differentiation of myofibroblasts, respectively. CD31-positive vessel counts were markedly increased in KB131-treated mice compared with vehicle-treated mice on days 5, 7, and 10 (Fig. 1F), and KB131 treatment led to an increase in α-SMA positive area on days 5 and 7 (Fig. 1G). Next, we examined the production of growth factors at wound sites after KB131 treatment. As shown in Fig. 1H, there was significantly increased synthesis of EGF, VEGF, bFGF, and TGF-β1 in KB131-treated mice at several time points.

### Enhanced accumulation of leukocytes by topical administration of heat-killed KB131

**Figure 2:**
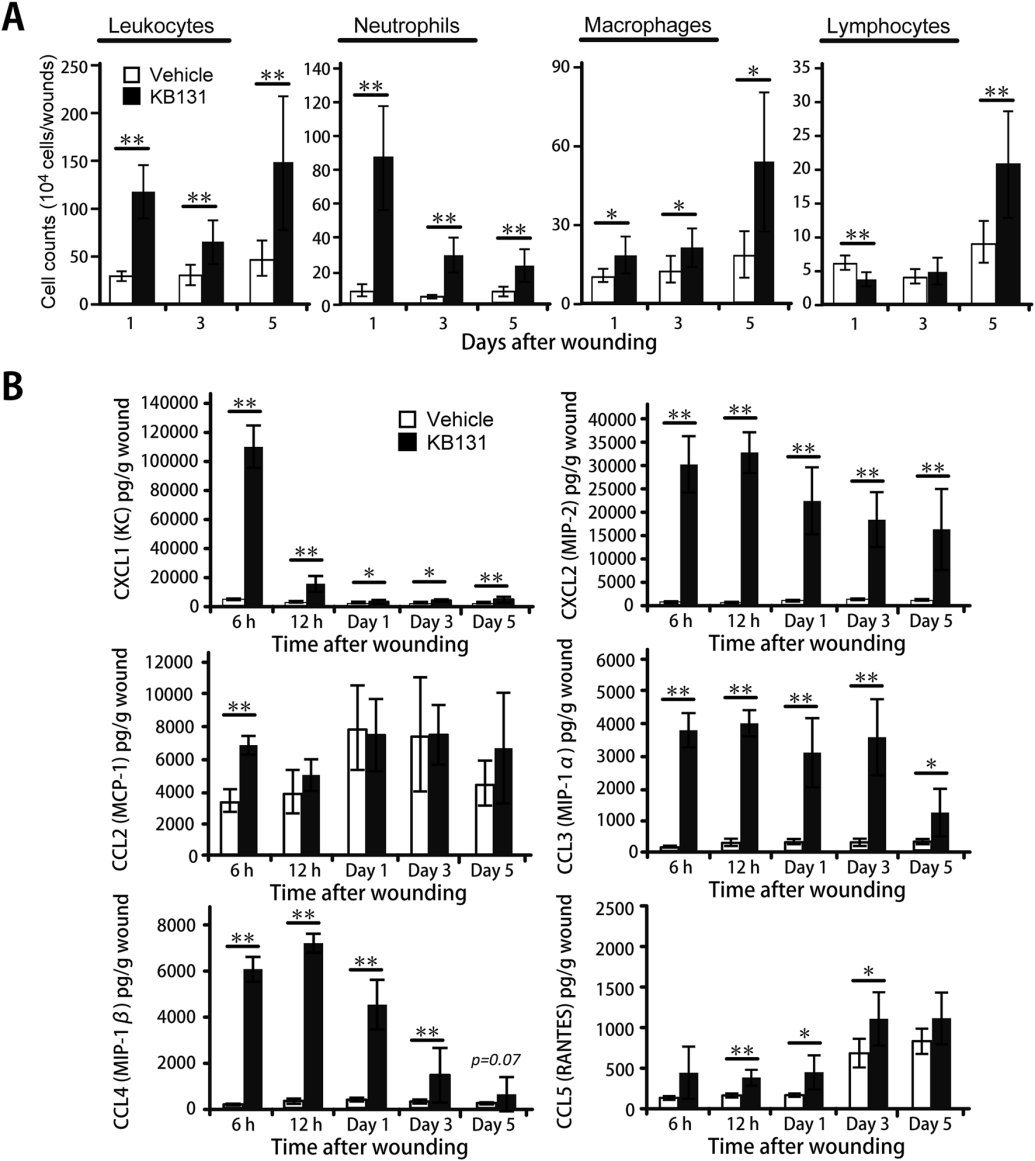
Effects of topical administration of heat-killed KB131 on accumulation of leukocytes and synthesis of cytokines and chemokines. (**A**) The numbers of leukocytes, neutrophils, macrophages, and lymphocytes in the wound tissue were analyzed by flow cytometric analyses (n = 6 mice per group). (**B**) Production of CXCL1 (KC), CXCL2 (MIP-2), CCL2 (MCP-1), CCL3 (MIP-1a), CCL4 (MIP-1b), and CCL5 (RANTES) in the wound tissues at 6 h, 12 h, and days 1, 3, and 5 after wounding (n = 5 mice per group). Each column represents the mean ± SD. *p < 0.05, **p < 0.01. *h* hour; *WT* wild type.

To define the role of inflammatory leukocytes during the healing process after KB131 treatment, leukocytes were isolated from wound sites, and the proportions of neutrophils (CD45^+^CD11b^+^Ly6G^+^), macrophages (CD45^+^CD11b^+^F4/80^+^ cells), and lymphocytes (CD3^+^, NK1.1^+^, TCRγδ^+^, or B220^+^) among them were evaluated by flow cytometry. The gating strategy used in the analysis of the leukocyte fraction and representative scatter plots is shown in Supplementary Fig. 1 and our previous study^20^. In mice treated with KB131, neutrophil counts were markedly increased in the early phases and gradually decreased, whereas that of macrophages and lymphocytes were found to be incrementally increased at day 5 after KB131 treatment compared with vehicle-treated mice (Fig. 2A). As shown in Fig. 2B, the synthesis of neutrophil chemokines, such as CXCL1 and CXCL2, were found to be significantly increased at 6 h after wounding, and the former was quickly reduced whereas the latter was gradually decreased. The kinetics of CXCL2 synthesis was parallel to that of neutrophil accumulation at the wound sites. In addition, the CCL3 and CCL4 syntheses were also markedly higher in the KB131-treated mice than in the vehicle-treated mice from the early inflammatory phase to the proliferative phase whereas CCL2 synthesis was significantly increased only at 6 h in KB131-treated mice. In contrast, the synthesis of CCL5 was found to be incrementally increased up to day 5, which was significantly higher at 12 h, day 1, and day 3 in the KB131-treated mice than in the vehicle-treated mice, in parallel with the accumulation of macrophages at the wound sites.

### Augmented accumulation of macrophages at the wound sites in KB131-treated mice

**Figure 3:**
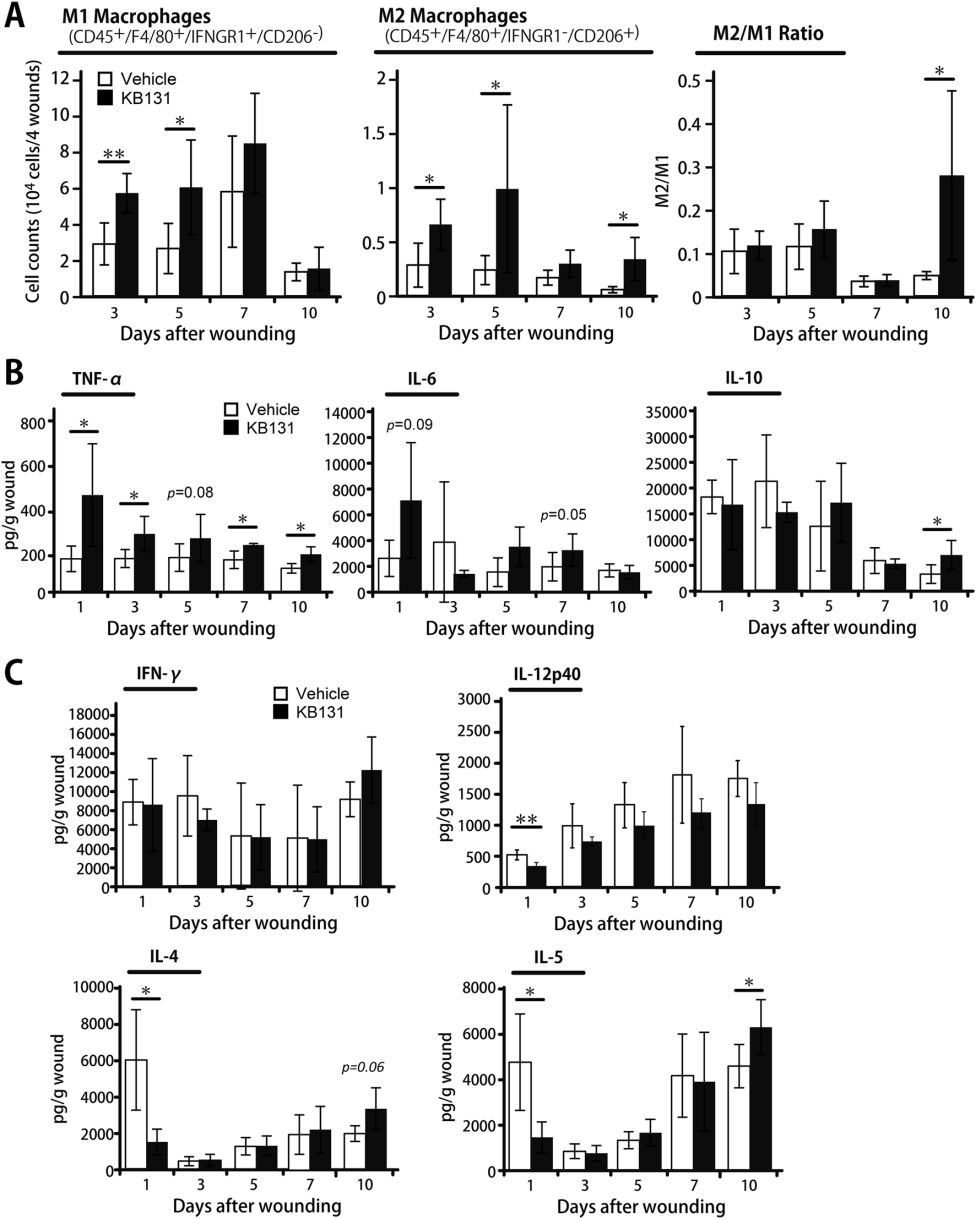
Effects of topical administration of heat-killed KB131 on macrophage phenotype and synthesis of M1 and M2 macrophage–associated cytokines. (**A**) The number of M1 and M2 macrophages by flow cytometric analysis (n = 6 mice per group), and ratio of M2 to M1 in the wound tissues. (**B**) Production of TNF-α, IL-6, and IL-10 in the wound tissues at days 1, 3, 5, 7, and 10 after wounding (n = 5 mice per group). (**C**) Production of IFN-γ, IL-12p40, IL-4 and IL-5 in the wound tissues at days 1, 3, 5, 7, and 10 after wounding (n = 5 mice per group). Each column represents the mean ± SD. *p < 0.05, **p < 0.01. *TNF* tumor necrosis factor.

In wound healing, macrophages are well known to play a critical role in facilitating the inflammatory response–proliferation phase transition through the generation of a variety of cytokines and chemokines^6,19^, and a proper switch from proinflammatory M1 type to anti-inflammatory and immunoregulatory M2 type accelerates the healing processes^6^. The gating strategy used to analyze M1 and M2 macrophages using flow cytometry as well as representative scatter plots are shown in Supplementary Fig. 2. As shown in Fig. 3A, in the KB131-treated group, the number of M1 macrophages (CD45^+^F4/80^+^IFNGR1^+^CD206^-^ cells) was significantly higher than in the vehicle-treated group at days 3 and 5 whereas the number of M2 macrophages (CD45^+^F4/80^+^IFNGR1^-^CD206^+^ cells) began to increase on day 3 and remained at a high level until day 10 and there was a marked increase in the ratio of M2 to M1 compared with the vehicle-treated group on day 10 after wounding. Next, examination of the proinflammatory cytokines in the wounded tissues showed that a higher level of TNF-α production was detected at days 1, 3, 7, and 10 in KB131-treated mice than in vehicle-treated mice whereas IL-6 synthesis was almost comparable between the two groups, although a tendency for higher production was detected at day 1, 5, and 7 in KB131-treated mice. We also confirmed that the production of anti-inflammatory cytokine IL-10 was significantly increased at day 10 by KB131 treatment whereas no significant difference was detected between the two groups from day 1 to 7 (Fig. 3B).

On the other hand, as shown in Fig. 3C, the synthesis of Th2 cytokines, IL-4, and IL-5, was significantly lower at day 1 and higher at day 10 in the KB131-treated group than in the control group whereas in the synthesis of Th1 cytokine, IL-12p40 levels were significantly higher on day 1, and IFN-γ, there was no significant difference between the two groups at all time points.

### Attenuated wound healing in CARD9-KO mice after heat-killed KB131 administration

**Figure 4:**
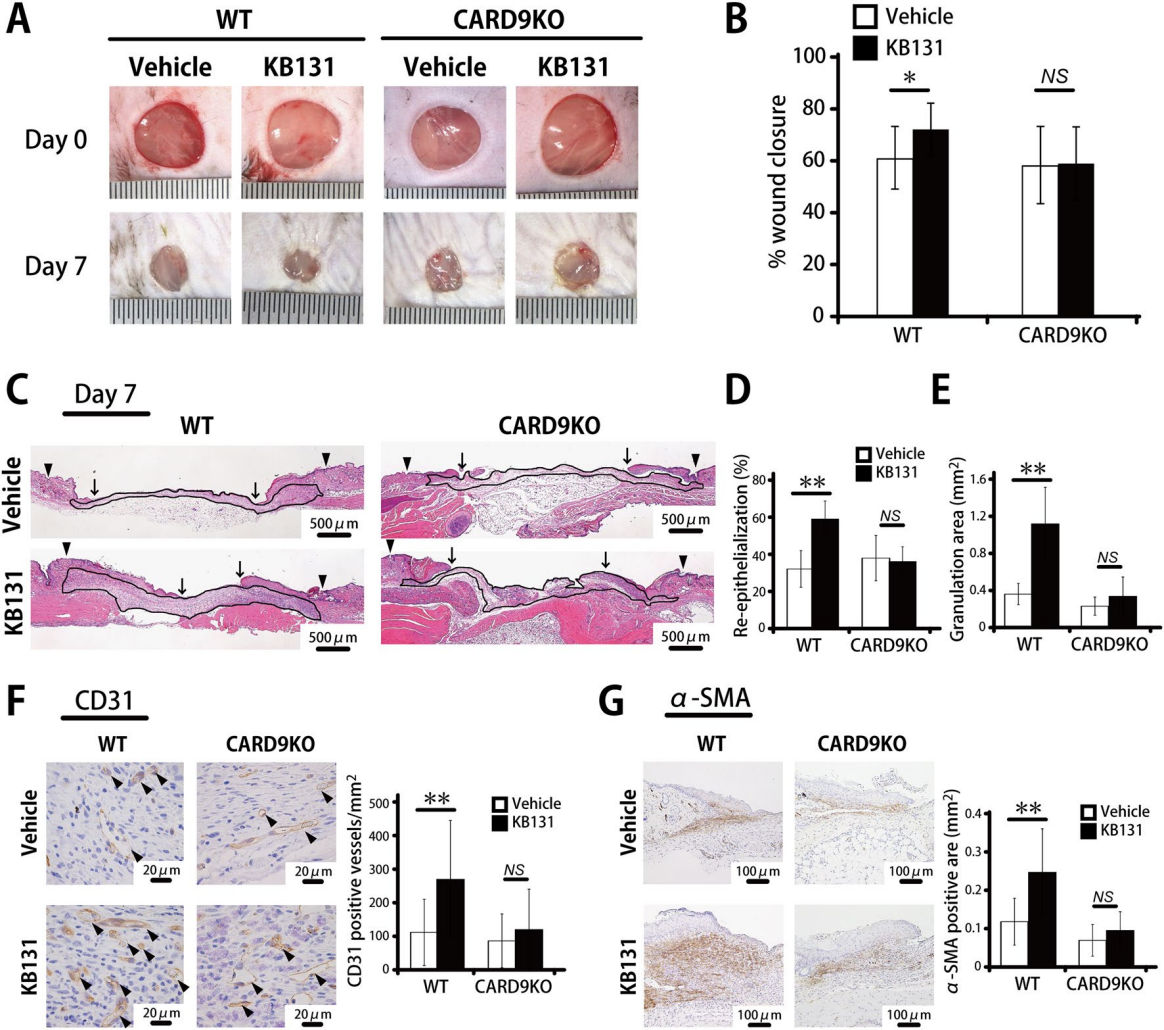
Effects of CARD9 deficiency on heat-killed KB131-treated wound healing. Wounds were created on the backs of WT and CARD9-KO mice, and immediately after wounding, KB131 or vehicle control was applied to the base of the wounds. (**A**) Representative photographs of wounds on days 0 and 7. (**B**) Percentage of wound closure was evaluated on day 7 (n = 20 wounds per group). (**C**) Representative histological views of the skin wounds. Arrowheads and arrows indicate the re-epithelialized leading edges and the original wound edges, respectively. (**D**) The re-epithelialization ratio on day 7 are shown (n = 10 wounds per group). (**E**) The granulation tissue area per mm^2^ on day 7 after wounding (n = 10 wounds per group). (**F**) The number of vessels stained with anti-CD31 antibody on day 7. Arrowheads indicate CD31-positive vessels. The vascular density per mm^2^ was determined by counting the positive vessels (n = 10 wounds per group). (**G**) The myofibroblast accumulated area per mm^2^ stained with α-SMA antibody on day 7 (n = 10 wounds per group). Each column represents the mean ± SD. *p < 0.05, **p < 0.01. *NS* not significant.

Previously, we demonstrated that CARD9-mediated signaling is involved in wound healing through macrophage accumulation^19,20^. Therefore, to address the possible contribution of this signaling molecule, we compared the wound healing process between wild-type (WT) and CARD9-KO mice after KB131 treatment. As shown in Fig. 4A,B, KB131 treatment demonstrated a significant promotion in wound closure on day 7 compared with the vehicle treatment in WT mice whereas these responses to KB131 treatment were canceled in CARD9-KO mice. Similar results were obtained when the re-epithelialization rate, granulation area, CD31-positive vessels, and α-SMA-positive area were examined (Fig. 4C–G). In contrast, when the effect of a lack of MyD88, another signaling molecule triggered by TLRs, was examined, wound closure was significantly promoted by KB131 treatment in MyD88KO mice and in WT mice (Supplementary Fig. 3).

## Discussion

In this study, we showed that wound healing was accelerated by the topical administration of heat-killed *L. plantarum* KB131, which was accompanied by an increase in M2 macrophages from the early inflammatory phase to the proliferative phase. In addition, the acceleration of wound healing responses by KB131 treatment was canceled under conditions lacking CARD9 expression. To the best of our knowledge, there have been no previous reports on the contribution of CARD9 to the effects of heat-killed *L. plantarum* on skin wound healing and increase of M2 macrophages. In addition, we confirmed that acceleration of wound healing after administration of heat-killed *L. plantarum* KB131 was observed in both male mice and female mice.

In previous investigations, viable LAB was frequently used to examine potential accelerators of skin wound healing^8,9^. Recently, however, several researchers in nutrition and microbial physiology have focused on heat-killed, rather than viable, LAB, because of safety concerns related to the risks of using live microorganisms, such as systemic infections and antimicrobial-resistant bacteria^11,12^. In the present study, we found that the administration of heat-killed *L. plantarum* KB131 resulted in accelerated wound closure, which was accompanied by the promotion of re-epithelialization, granulation, neovascularization, and accumulation of α-SMA-positive myofibroblasts, as well as an increase in M2 macrophages and synthesis of growth factors. It has been reported that multiple factors, such as TNF-α and EGF, contribute to the re-epithelialization^23,24^, and several growth factors, such as VEGF, bFGF, and TGF-β1, are involved in the angiogenesis and expression of α-SMA in granulation tissues after wounding^25,26^. In the present study, the early phase TNF-α production and the late phase EGF synthesis were found to be increased, and the production of other growth factors was also upregulated by the topical administration of KB131. Thus, these factors likely contribute to the promotion of wound healing processes.

Although little is known about the effects of heat-killed LAB on the inflammatory responses, including the kinetics of M1 and M2 macrophages, it has been reported that early phase TNF-α synthesis was potentiated after *E. faecalis* KH2 administration^13^ and that neutrophil myeloperoxidase (MPO) activity was ameliorated in the proliferative phase after *L. chungangensis* CAU 1447 treatment^15^. In the current study, the topical administration of heat-killed *L. plantarum* KB131 caused accelerated wound repair that was accompanied by the increased accumulation of neutrophils and M1 macrophages in the early phase and also by the increase of M2 macrophages from the early inflammatory phase to the proliferative phase. Neutrophils are well known to produce a variety of cytokines and growth factors, including TNF-α^24^ and VEGF^27^, suggesting that these cells may play a part in the orchestrated polarization of macrophages towards a reparative M2 phenotype in tissue repair processes^28^. In our model, KB131 administration led to accelerated wound repair, which was observed together with an increase of M2 macrophages and M1 macrophages as well as a significant increase in the ratio of M2 to M1 macrophages on day 10, when synthesis of IL-5 and IL-10 was significantly increased. Thus, the regulation of inflammatory responses caused by KB131 administration may contribute to the generation of optimal conditions for switching macrophage polarization and promoting better wound healing processes as a result.

Macrophages not only eliminate microbes and tissue debris but also initiate tissue remodeling, such as formation of granulation tissues through the secretion of several growth factors, such as VEGF^3,29^. Recent investigations have revealed that the proper switch of macrophages from proinflammatory M1 to anti-inflammatory M2 phenotypes is required for efficient wound healing responses^6^. Although the involvement of heat-killed *L. plantarum* in M1/M2 macrophage polarization at wound sites has not been reported, Kim et al.^30^ recently demonstrated that *L. plantarum-*derived extracellular vesicles, such as lipid bilayer-enclosed nanosized vesicles, promoted the differentiation of human monocytic THP-1 cells toward the M2 phenotype in in vitro experiments^30^. In addition, the oral administration of viable *L. plantarum* CLP-0611 was reported to increase M2 macrophages expressing IL-10, arginase I, and CD206, which was likely involved in the amelioration of 2,4,6-trinitrobenzene sulfonic acid (TNBS)-induced mice colitis, although the kinetics of macrophages was not examined in detail^31^. In the present study, we observed that KB131-treated mice underwent accelerated wound repair that was associated with a significant increase in M2 macrophages from day 1 to day 10 together with a marked increase in the ratio of M2 to M1. Importantly, these alterations were not observed in control mice treated with vehicle. Thus, the current results suggest that accelerated wound healing by heat-killed KB131 may involve the more appropriate switching of macrophage phenotypes.

In the present study, the topical administration of KB131 induced the early synthesis of CXCL1 (KC) and CXCL2 (MIP-2), leading to acute phase neutrophil accumulation at the wound tissues. The efficient recruitment of neutrophils to injured skin is an essential innate immune response for the healing process of skin wounds^24,32,33^, and CXCL1 and CXCL2/CXC chemokine receptor 2 (CXCR2) signaling play a critical role in the recruitment of these inflammatory cells. During wound healing, keratinocytes and endothelial cells were reported to induce the synthesis of CXCR2 and trigger CXCL1 and CXCL2/ CXCR2 signaling, leading to enhancement of wound healing responses, such as re-epithelialization^34^ and neovascularization^35^. Taken together with these previous findings, the current results suggest that CXCL1 and CXCL2 synthesis may be involved in the acceleration of re-epithelialization and neovascularization at the skin wound tissues after treatment with heat-killed *L. plantarum* KB131.

CCL5 is well known as a chemokine that recruits several immune cells, including M2 macrophages, at inflammatory sites^36,37^ and also plays a critical role during wound healing^38^. Previously, it was reported that the topical administration of CCL5 at wound sites improved wound healing in the later phase, such as day 7 post-wounding^39^. In addition, Ishida et al. reported that the interaction of CCL5 with its receptor, CCR5, was an important process for inducing neovascularization in a mouse wound healing model^40^. In the present study, the increase in M2 macrophages was well correlated with the kinetics of CCL5 synthesis after KB131 administration. Thus, the improved neovascularization and formation of granulation area might involve the increase in M2 macrophages followed by CCL5 synthesis after KB131 administration.

Microorganisms possess a variety of pathogen molecular patterns that lead to the activation of immune cells via interaction with relevant PRRs, which trigger signaling pathways mediated by adaptor molecules, including MyD88 and CARD9^41,42^. Previous investigations reported the involvement of MyD88-mediated signaling via some TLRs in the LAB-induced protection of intestinal epithelium from radiation injury^43^. Therefore, we first examined how genetic disruption of MyD88 affected the upregulated wound healing by KB131. The results indicated that this bacterium accelerated wound healing even without MyD88 expression (Supplementary Fig. 3), suggesting the irrespective role of this adaptor molecule. In contrast, in previous investigations using *Lactobacillus* spp., interaction of the Surface (S)-layer from *L. brevis* with Mincle was found to result in the synthesis of cytokines from bone marrow-derived cells, and bone marrow-derived cells derived from Mincle^–/–^ and CARD9^-/-^ mice were reported to produce a reduced level of IL-10 compared to those from WT mice, with the same stimulation^44^. In addition, Yoshikawa and co-workers demonstrated that *L. paracasei* KW3110 was easily incorporated by RAW 264.7 cells (macrophage cell line) through Dectin-2-mediated phagocytosis, which led to the enhanced production of IL-10^18^. These reports only observed IL-10 production by bone marrow-derived cells or RAW cells in vitro, however. In the present study, in contrast, we found that the accelerated wound healing responses by the topical administration of heat-killed *L. plantarum* KB131 were, as shown by the enhanced re-epithelialization and formation of granulation area, canceled in CARD9KO mice. Moreover, to identify the upstream receptor of this adapter molecule, we examined if this bacterium triggered the signaling pathway via Dectin-1, Dectin-2, and Mincle using reporter cells expressing either receptor together with the GFP gene. Unfortunately, we did not obtain positive results indicating the involvement of either receptor tested. Taken together, the current results raise the possibility that other CLRs may be involved in the KB131-induced acceleration of wound healing responses. Further investigations are required to clarify these issues.

In conclusion, the current study suggested that the topical administration of heat-killed *L. plantarum* KB131 immediately after wounding may lead to appropriate responses at the proliferative phase, which was accompanied by the increase in M2 macrophages, and that CARD9-mediated signaling may be deeply involved in the progression of these responses. These points are summarized in Supplementary Fig. 4. Recently, heat-killed probiotics have attracted increasing attention related to their health benefits, because it has gradually become clear that the immunostimulatory effects of heat-killed LAB are nearly equivalent or more potent than those of viable cells^12^. For example, Jin et al. reported that heat-killed *L. plantarum* Zhang-LL was more effective than that of live bacteria in improving ulcerative colitis caused by dextran sulfate sodium in rats^45^. Thus, the present study significantly enhances our understanding of the immune responses for wound repair accelerated by the administration of heat-killed LAB.

## Materials and methods

### Ethical statement

This study was performed in strict accordance with the Fundamental Guidelines for Proper Conduct of Animal Experiments and Related Activities in Academic Research Institutions under the jurisdiction of the Ministry of Education, Culture, Sports, Science and Technology in Japan, 2006. All experimental procedures involving animals followed the Regulations for Animal Experiments and Related Activities at Tohoku University, Sendai, Japan and were approved by the Institutional Animal Care and Use Committee at Tohoku University. All methods were performed in accordance with the relevant guidelines and regulations, and all efforts were made to minimize the suffering of the animals. The study was carried out in compliance with the ARRIVE guidelines (https://arriveguidelines.org/).

### Animals

CARD9 gene–disrupted knockout (KO) mice were generated and established as described previously^46^, and backcrossed to C57BL/6 J mice for more than eight generations. MyD88KO mice, established as described previously^47^, and backcrossed to C57BL/6 J mice, were purchased from OrientalBioService, Inc. (Kyoto, Japan). Wild-type (WT) C57BL/6 J mice, purchased from CLEA Japan (Tokyo, Japan), were used as a control. Male mice at 7 to 10 weeks of age were used in all experiments shown in Figs. 1, 2, 3 and 4. For the results shown in Supplementary Fig. 3, female mice were used in half the experiments, because there were not sufficient numbers of MyD88KO male mice for all analyses. Food and water were available ad libitum. All mice were kept under specific pathogen-free conditions in the Institute for Animal Experimentation, Tohoku University Graduate School of Medicine (Sendai, Japan). All experimental protocols described in this study were approved by the Ethics Review Committee for Animal Experimentation of Tohoku University. All experiments were performed under anesthesia, and all efforts were made to minimize the suffering of the animals.

### Wound creation and tissue collection

Mice were anesthetized by intraperitoneal injection of 40 mg/kg sodium pentobarbital (Somnopentyl, Kyoritsu Seiyaku Corporation, Tokyo, Japan), and sustained by inhalation anesthesia of isoflurane (Isoflurane, Mairan Pharma, Osaka, Japan). The dorsal hair was shaved to fully expose the skin, which was then rinsed with 70% ethanol. Four full-thickness wounds extending to the panniculus carnosus were created on each mouse using a 6-mm-diameter biopsy punch (Biopsy Punch, Kai Industries Co., Ltd., Gifu, Japan) under sterile conditions and covered with a polyurethane film (Tegaderm Transparent Dressing, 3 M Health Care, St. Paul, MN, USA) and an elastic adhesive bandage (Hilate, Iwatsuki, Tokyo, Japan) for an occlusive dressing. At various time points, mice were sacrificed by overdose of isoflurane, and the wound tissue was collected by excising a 1-cm-square section of skin using scissors and a surgical knife. The tissue was then processed for histopathological analysis and measurement of cytokine concentrations.

### Preparation of heat-killed *Lactobacillus plantarum* KB131

*L. plantarum* KB131 (International Patent Organism Depositary, Japan, NITE BP-03375) was stored at Bio-Lab Co., Ltd. *L. plantarum* KB131 was grown aerobically overnight at 37 °C in MRS broth (Difco, Detroit, MI, USA) and washed with distilled water, followed by centrifugation at 10,000×*g* for 3 min. The bacterial suspension in distilled water was heated at 105 °C for 30 min using an autoclave (HV-25IILB; Hirayama Manufacturing Corp., Saitama, Japan). Based on the recent reclassification of LAB, *L. plantarum* belongs to the *Lactiplantibacillus* genus^48^. We confirmed using species-specific primers that KB131 belonged to *L. paraplantarum*^49^. Wounds were created in accordance with the method described above. Immediately after wounding, a 5 μL suspension of heat-killed *L. plantarum* KB131 (125 μg/μg) or distilled water as a vehicle control was applied to the base of the wounds in mice.

### Measurement of the wound area

Morphometric analysis was performed on digital images using a digital camera (G800, Ricoh, Tokyo, Japan). After the wounds were created, photographs were taken of each wound before dressing. At various time points, the polyurethane films were gently removed from the sacrificed mice, and the wounds were photographed. The wound area was quantified by tracing its margin and calculating the pixel area using AxioVision imaging software, Release 4.6 (Carl Zeiss Micro Imaging Japan, Tokyo, Japan). Wound healing was evaluated as the percent wound closure, which was calculated using the following formula: % wound closure = (1 − wound area at the indicated time point/wound area on day 0) × 100.

### Histopathology and immunohistochemistry

The wounded tissues were fixed with 4% paraformaldehyde-phosphate buffer solution and embedded in paraffin after caudocranial dissection, as previously described^20,21,50^. Sections were harvested from the central portion of the wound and stained with hematoxylin–eosin (HE) according to the standard method. The extent of re-epithelialization of each wound was measured in these HE-stained sections by measuring the distance from the normal wound margin to the edge of the epithelium. The re-epithelialization index was determined based on the percentage of new epithelium present in the total wound. Granulation area was determined on HE-stained sections. For immunohistochemistry, the sections were stained with anti-CD31 antibody (1:600 dilution; R&D Systems, Minneapolis, MN, USA), anti-α-smooth muscle actin (α-SMA) antibody (1:300 dilution; Dako, Santa Clara, CA, USA), or anti-F4/80 antibody (1:100 dilution; BioLegend, San Diego, CA, USA) after endogenous peroxidase and nonspecific binding were blocked. They were then incubated with peroxidase-conjugated secondary antibodies (Histofine® Simple Stain MAX-PO, Nichirei Bioscience, Tokyo, Japan). Control sections were treated with non-immune IgG in place of any of the first antibodies. The vascular density in granulation tissue and the number of macrophages in each of five random fields (each 0.03 mm^2^) were determined by counting the number of CD31-positive vessels and the number of F4/80-positive cells, respectively. The amount of myofibroblasts was determined by calculating the area of α-SMA-positive cells.

### Measurement of cytokine, chemokine, and growth factor concentrations

The wounded tissues were homogenized with saline solution, and the concentration of cytokines and chemokines in the supernatants was measured using appropriate enzyme-linked immunosorbent assay (ELISA) kits (BioLegend, San Diego, CA, USA, for TNF-α, IL-4, IL-5, IL-6, IL-10, IFN-γ, IL-12p40; R&D Systems, Minneapolis, MN, USA, for CXCL1, CXCL2, CCL2, CCL3, CCL4, CCL5, bFGF, TGF-β1, VEGF, and EGF). The results were expressed as the values per wound.

### Preparation of leukocytes in the wound tissue

Mice were sacrificed at days 1, 3, or 5 after wound creation. The wound tissues were excised and teased apart using stainless-steel mesh in RPMI 1640 medium (Sigma-Aldrich, St. Louis, MO, USA) supplemented with 10 mM HEPES, 10% fetal calf serum (FCS) (BioWest, Nuaillé, France), 0.2 mg/mL Liberase TL, 2.5 mg/mL collagenase, 0.1 mg/mL DNase, and 2.0 mg/mL Dispase (Sigma-Aldrich). They were incubated for 2 h at 37 °C with vigorous shaking. After incubation, the tissue fragments and most dead cells were removed by passing the cells through a 70-μm cell strainer (BD Falcon, Bedford, MA, USA). After centrifugation, the cell pellet was resuspended in 4 ml of 40% Percoll (Pharmacia, Uppsala, Sweden) and layered onto 4 ml of 80% Percoll. After centrifugation at 1800 rpm for 20 min at 20 °C, the cells at the interface were collected, washed three times with staining buffer, and counted using a hemocytometer.

### Analysis of leukocyte fraction using flow cytometric analysis

The cells obtained from the wounded tissues were stained with 7-aminoactinomycin D (7-AAD) (BioLegend), Pacific Blue-anti-CD45 monoclonal antibody (mAb) (clone 30-F11, BioLegend), APC-anti-CD11b mAb (clone M1/70, BioLegend), APC/Cy7-anti-Ly6G mAb (clone 1A8, BioLegend), PE-anti-F4/80 mAb (clone BM8, BioLegend), FITC-anti-CD3ε mAb (clone 145-2C11, BioLegend), FITC-anti-NK1.1 mAb (clone PK136, BioLegend), FITC-anti-T-cell receptor γδ (TCRγδ) mAb (clone GL3, BioLegend), and FITC-anti-CD45R/B220 mAb (clone RA3-6B2, BioLegend). Isotype-matched irrelevant IgG was used for control staining. To investigate macrophage subtypes, the cells were further stained with biotin-conjugated anti-mouse CD119 (IFN-γ R α chain) mAb (clone 2E2, BioLegend), followed by staining with APC/Cy7 Streptavidin (BioLegend). In addition, CD206 was stained according to the manufacturer’s methods. Briefly, leukocytes from wounded tissues were incubated in the presence of Cytofix/Cytoperm (BD Biosciences, Franklin Lakes, NJ), washed twice in BD perm/wash solution, and stained with APC-anti-CD206 antibody (clone C068C2; BioLegend) or control rat IgG. Neutrophils and macrophages were identified as CD45^+^CD11b^+^Ly6G^+^ cells and CD45^+^CD11b^+^F4/80^+^ cells, respectively. Lymphocytes were identified as CD45^+^ cells expressing CD3ε, NK1.1, TCRγδ, or B220. M1-type and M2-type macrophages were identified as CD45^+^F4/80^+^IFNGR1^+^ CD206^-^ cells and CD45^+^F4/80^+^ IFNGR1^-^CD206^+^ cells, respectively. The stained cells were analyzed using a BD FACS Canto II flow cytometer (BD Bioscience, Franklin Lakes, NJ, USA). The number of neutrophils, macrophages, and lymphocytes was estimated by multiplying the total leukocyte number by the proportion of each fraction.

### Statistical analysis

Data are expressed as the mean ± standard deviation (SD). Data analysis was performed using Welch’s *t*-test to compare two experimental groups, and a one-way analysis of variance (ANOVA) with post hoc Dunnett’s or Tukey–Kramer’s honestly significant difference (HSD) test was used for more than three experimental groups. A *p*-value less than 0.05 was considered to indicate statistical significance.

### Supplementary Information

Supplementary Figures.

## Data Availability

The data that support the findings of this study are available from the corresponding author upon reasonable request.
