# Supplementary material for: Cutaneous wound healing promoted by topical administration of heat-killed Lactobacillus plantarum KB131 and possible contribution of CARD9-mediated signaling
Source: Sci Rep. 2023 Sep 23;13:15917. doi: 10.1038/s41598-023-42919-z (PMC10517988; doi:10.1038/s41598-023-42919-z)
Supplement: Supplementary file 1 — Supplementary Figures. [file 41598_2023_42919_MOESM1_ESM.pdf]

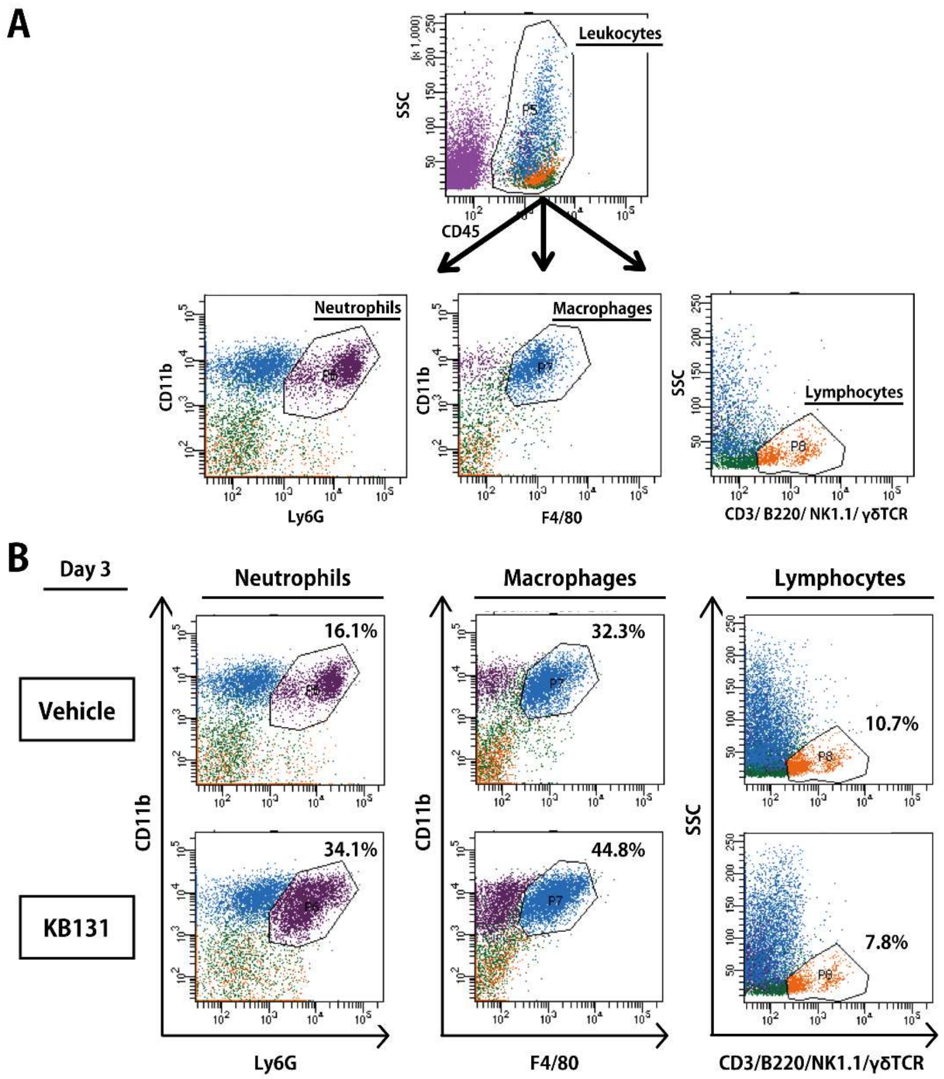

Supplementary Figure 1. Gating strategy for analysis of the leukocyte fraction using flow cytometry and representative scatter plots. The cells obtained from the wound tissues were stained with Pacific blue-anti-CD45 monoclonal antibody (mAb), APC-anti-CD11b mAb, APC/ Cy7-anti-Ly6G mAb, PE-anti-F4/80 mAb, FITC-anti-CD3ε mAb, FITC-anti-NK1.1 mAb, FITC-anti-T-cell receptor γδ (TCRγδ) mAb, and FITC-anti-CD45R/B220 mAb. (A) Neutrophils and macrophages were identified as CD45+CD11b+Ly6G+ cells and CD45+CD11b+F4/80+ cells, respectively. Lymphocytes were identified as CD45+ cells expressing CD3, NK1.1, TCRγδ, or B220. The stained cells were analyzed using flow cytometry. (B) Flow cytometry plot representation in each group.

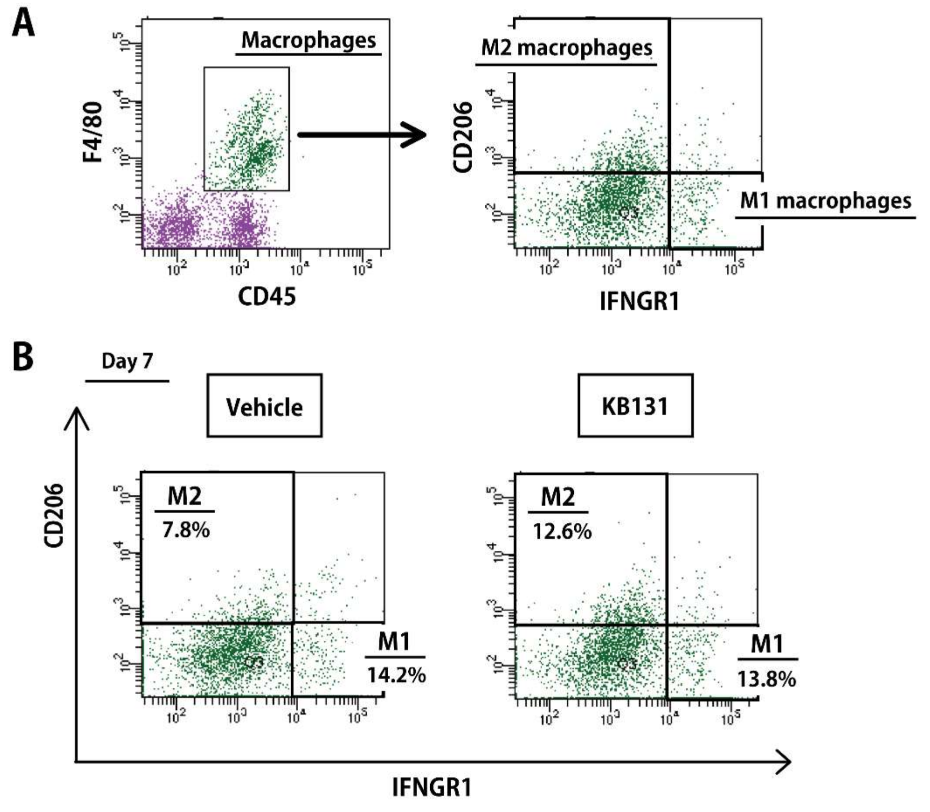

Supplementary Figure 2. Gating strategy for the analysis of M1 and M2 macrophages using flow cytometry and representative scatter plots.
We identified M1/M2 macrophages by gating on F4/80-positive macrophages and analyzing their IFNGR1 (M1 macrophage marker) or CD206 (M2 macrophage marker) expression. (A) M1-type and M2-type macrophages were identified as CD45+F4/80+IFNGR1+CD206- cells and CD45+F4/80+IFNGR−CD206+ cells, respectively. (B) Flow cytometry plot representation in each group.

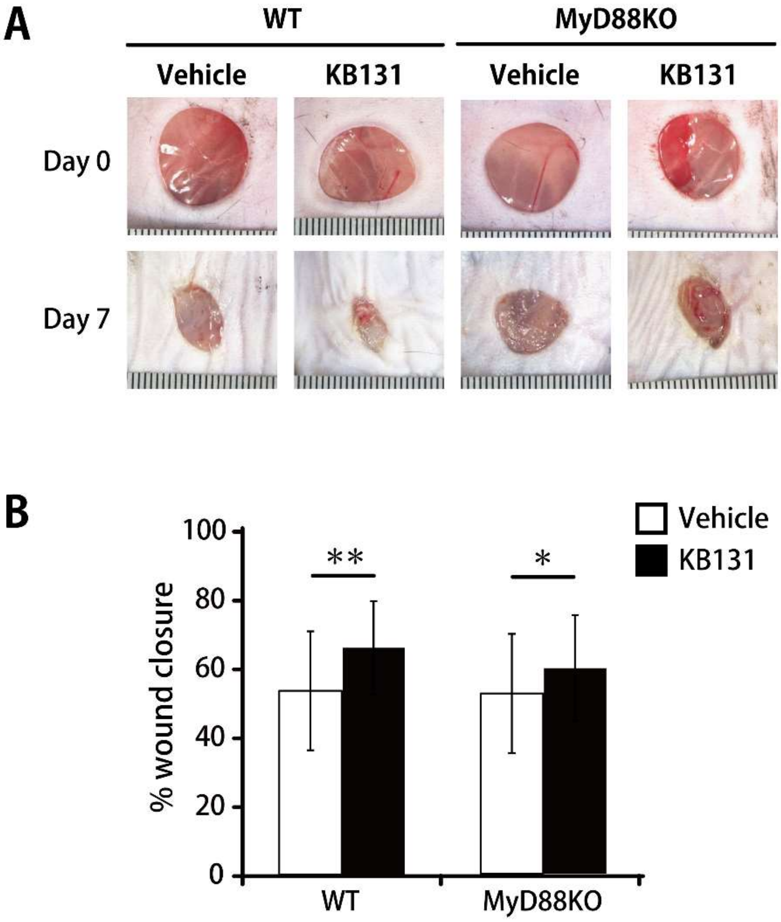

Supplementary Figure 3. Effects of MyD88 deficiency on heat-killed KB131-treated wound healing. Wounds were created on the backs of WT and MyD88-KO mice. Immediately after wounding, KB131 or vehicle control was applied to the base of the wounds. (A) Representative photographs of wounds on days 0 and 7. (B) Percentage of wound closure was evaluated on day 7.

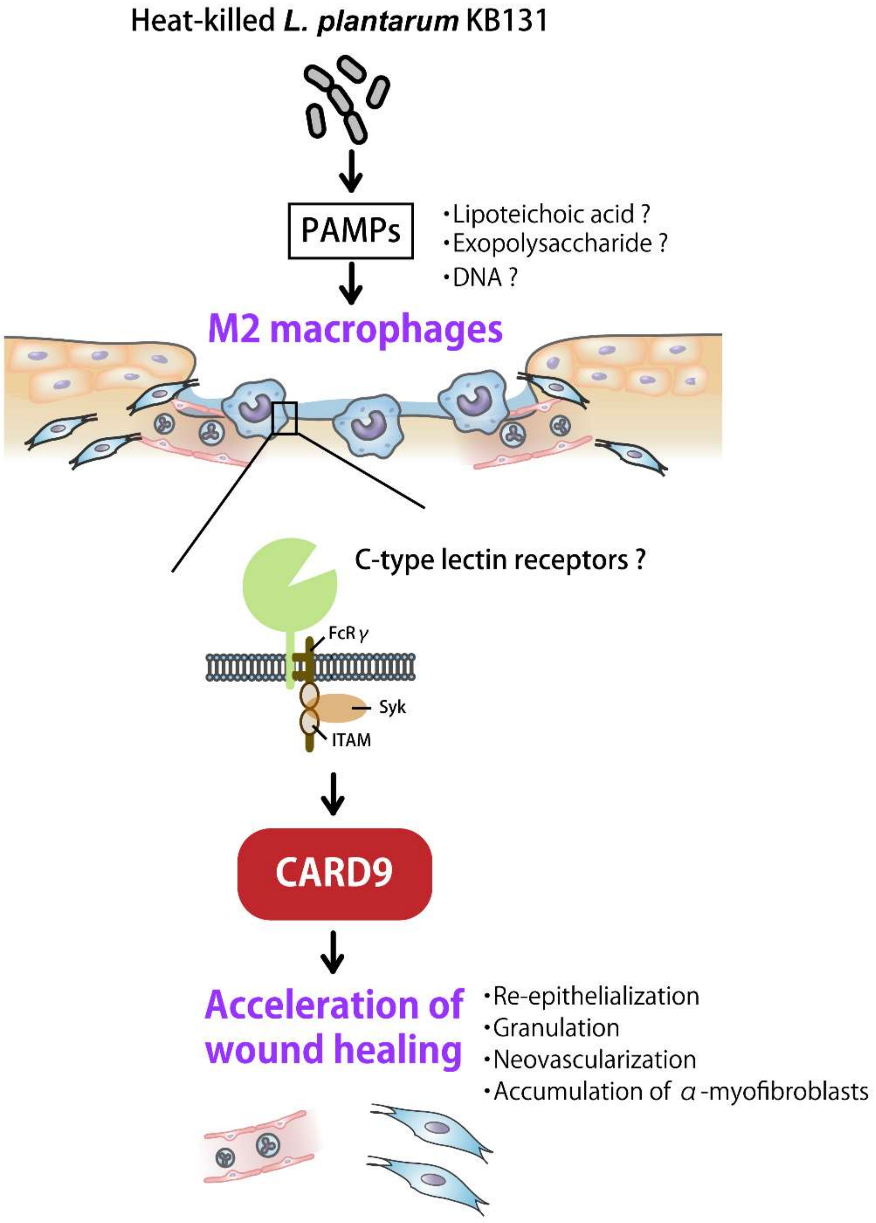

Supplementary Figure 4. Cutaneous wound healing promoted by topical administration of heat-killed Lactobacillus plantarum KB131 and possible contribution of CARD9-mediated signaling.
Topical administration of KB131 accelerates wound healing, accompanied by increased M2 macrophages, which suggests that CARD9 may be involved in these responses.
